# COVID-19 Phobia among Korean, Chinese, and Japanese students: An international comparative study

**DOI:** 10.1016/j.heliyon.2023.e15275

**Published:** 2023-04-07

**Authors:** Hocheol Lee, Fanlei Kong, Motoyuki Yuasa, Myo Nyein Aung, Yoshihisa Shirayama, Bo Zhao, Mahyeon Kim, Eun Woo Nam

**Affiliations:** a Yonsei Global Health Center, Yonsei University, Wonju, Gangwon-do, Republic of Korea; b Centre for Health Management and Policy Research, School of Public Health, Cheeloo College of Medicine, Shandong University, Jinan, 250012, China; c NHC Key Lab of Health Economics and Policy Research (Shandong University), Jinan, 250012, China; d Department of Global Health Research, Graduate School of Medicine, Juntendo University, Hongo 2-1-1, Bunkyo Ku, Tokyo 113-8421, Japan; e Department of Health Administration, Software Digital Healthcare Convergence College, Yonsei University, Wonju 26493, South Korea

**Keywords:** Fears, COVID-19 fear, Mental health, Pandemic, Young people

## Abstract

This study aimed to identify the characteristic differences and potential contributing factors of coronavirus disease 2019 (COVID-19) phobia between undergraduate and graduate students in Korea, Japan, and China. We used the online survey tool, we retained 460 responses from Korea, 248 responses from Japan, and 788 responses from China for analysis. We performed the statistical analysis; ANOVA F-test and Multiple linear regression. We visualized the results of these calculations using GraphPad PRISM 9. The mean COVID-19 phobia score was highest in Japan at 50.5 points. Psychological fear was identically prevalent in Japan and China, at an average of 17.3 points. Psychosomatic fear was highest in Japan at 9.2 points. Further, economic fear was highest in Korea at 13 points, whereas social fear was highest in China at 13.1 points. In Korea, COVID-19 phobia scores were significantly higher among women than in men. In Japan, COVID-19 phobia scores were significantly higher in individuals who complied with social distancing mandates. In China, a lack of previous experience with self-administered testing kits was associated with significantly lower phobia scores. Individuals who were avoiding crowded places had significantly higher scores in 3 countries. This implies that the students knew that it was necessary to comply with COVID-19 preventive behaviors to prevent infections. The findings of this study could be used as a reference when establishing an approach strategy to reduce COVID-19 phobia among Chinese, Japanese, and Korean students.

## Highlights

• First study comparing COVID-19 phobia among Korean, Japanese, and Chinese uni students during pandemic, 2022.
• COVID-19 phobia: Korea 48.1, Japan 50.5, China 49.9 points among uni students.
• Avoiding crowded places linked to higher phobia scales in Korean, Japanese, and Chinese uni students.

## 1 Introduction

At the end of December 2019, the coronavirus disease 2019 (COVID-19) was reported for the first time in the world in Wuhan, China, whereas the first cases in Korea and Japan were reported on January 19 and January 16, 2020, respectively. Subsequently, COVID-19 spread rapidly worldwide, and in March 2020, the World Health Organization (WHO) declared it a pandemic, considering its severity [1]. As of July 20, 2022, there have been more than 560 million confirmed cases and 6 million deaths reported across 229 countries.

To contain the spread of COVID-19, many countries adopted and implemented various quarantine policies, such as blockades in cities, social distancing, and immigration restrictions. With the prolongation of COVID-19, deterioration in mental health from the uncertainty of the future and social isolation, reductions in income, increased loneliness, and decreased social activity have been reported [2]. The economic recession caused by COVID-19 has led to higher rate of unemployment, instability of national economies, and poverty, which have negatively impacted the mental health of the population, including the younger generation [3]. Indeed, the literature on infectious disease and mental health posits that infectious disease outbreaks and preventive measures to combat their spread may worsen mental health across populations [4,5].

In particular, as COVID-19 is covered in global news, media outlets, and the internet, individuals may develop “headline stress disorder,” a type of stress disorder and fear [6]. The fear caused by COVID-19 is emerging as a novel social problem, with mental health (e.g., fear) being identified by the [7] as one of the nine major problems that need to be addressed in the post-COVID-19 era. Among these, the fear of COVID-19 among undergraduate students is a social problem; approximately a quarter of undergraduate students have reported feeling such fear [[8], [9], [10]]. In addition, the declaration of COVID-19 as a pandemic served as a potentially negative psychological impact that may be expressed as fear among university students [11]. Although policies were implemented in Korea, Japan, and China to protect students from COVID-19, such as shifting the delivery of lectures from in-person to remote classes and suspending certain courses, 24.9% of university students experienced fear from COVID-19 [12].

Korea has been gradually returning to normal life with the implementation of the “Living with COVID-19” policy announced in November 2021, along with the gradual easing of restrictions in universities to return to pre-COVID-19 activities, such as re-opening of student residences, expansion of in-person classes, lifting of restrictions on classroom capacities, and hosting of festivals. Nevertheless, according to statistics presented by the Ministry of Health and Welfare of South Korea, feelings of depression, called Corona Blue, increased six-fold compared with pre-pandemic levels [13]. Furthermore, university students are against the “Living with COVID-19” policy, claiming that it is premature. To reduce such fears surrounding COVID-19 among university students, the Korean Ministry of Education implemented the University Student Mental Health Support Program, which operates a psychological support call center. However, its effectiveness has not been examined. Meanwhile, in China, although the government attempted to strengthen quarantine policies among university students with their “Zero COVID” policy, the plan was withdrawn owing to opposition from the students. Such a decision was based on considerations for the mental health of university students, given the prolonged city lockdowns and restrictions on dining in restaurants and food orders, as well as the frequent COVID-19 testing. Nevertheless, the strict prevention measures by the Chinese government will continue to be enforced. The Chinese government has proposed the need to implement policies developed for the post-COVID-19 era, including the identification of factors that can reduce the fear of COVID-19 among university students. In Japan, the government has lifted restrictions on in-person classes, returning to normal in-person university lectures. Some experts have expressed concerns regarding the easing of such COVID-19 policies and the consequent potential spread of COVID-19. There is also a possibility that fear among university students regarding COVID-19 will increase.

With the prolongation of COVID-19, the Korean, Japanese, and Chinese governments must develop policies to manage “COVID blues” and fear among university students. The factors that contribute to fear surrounding COVID-19 among university students are to be identified and serve as a basis for newly developed policies. However, the literature on the detailed analysis of university students’ fear of COVID-19 in the three countries remains limited [12]. This study hypothesized that among these individual socio-ecological factors, personal behavior and knowledge affect mental health, such as fear of COVID-19. Moreover, different policies to respond to COVID-19 in each country may also affect individual fears.

Thus, this study aimed to identify the characteristic differences and potential contributing factors of COVID-19 phobia between undergraduate and graduate students in Korea, Japan, and China. Ultimately, we hope to offer evidence for establishing policies to reduce COVID-19 phobia among university students in each nation, as well as for the international community.

## 2 Method

### 2.1 Study design

This cross-sectional study aimed to identify the characteristics of COVID-19 phobia and its influencing factors prevalent among undergraduate and graduate students in Korea, Japan, and China.

### 2.2 Data sample and data collection

To calculate the sample size required for the regression analysis in this study, we performed an F-test and multiple linear regression analysis with a 95% confidence interval and 5% error range using G*Power 3.1. The results suggested that the sample size required for each country was 172. The survey periods were as follows: Korea, April 1 to 6, 2022; Japan, May 17 to June 6, 2022; and China, April 16 to May 16, 2022. Participants of the survey were recruited through public bulletin postings at universities to which the researchers from Korea, China, and Japan belong, and were allowed to participate voluntarily. The postings were written in the language of each country by the researchers in the respective country. The posts included an introduction to the study, a consent form, the data storage method, and the study objectives.

We created an online questionnaire using an online survey tool, initially developed in Korean and then translated to Japanese and Mandarin. We used the Google survey form in Korea and Japan and the online tool Wenjun in China. The surveys were conducted by sharing the Uniform Resource Locator (URL) generated by each online survey tool with participants who had agreed to participate in the survey. Upon elimination of responses that involved drop-outs, insincere responses, and missing values from the survey data. Responses were eliminated if they involved drop-outs, insincere responses, or missing values (data that were censored or duplicated) from the survey data. Finally, our study retained 460 responses from Korea, 248 responses from Japan, and 788 responses from China for analysis. The number of responses from each country met the minimum sample size required for data analysis.

### 2.3 Study instrument

Our survey tool was supplemented and developed for this study based on the COVID-19 Phobia Scale (C19P–S) by Ref. [14]. The C19P–S is a tool developed to measure COVID-19 phobia with verified validity and reliability. It covers four characteristics: psychological (6 items, 30 points), psychosomatic (5 items, 25 points), social (5 items, 25 points), and economic (4 items, 20 points). Our study utilized the COVID-19 phobia examined using the C19P–S as a dependent variable.

The independent variables of our study were as follows: sex (*male*, *female*), location of residence (*urban*, *rural*), experience using a self-administered test kit (*yes*, *no*), avoidance of crowded places (*yes*, *no*), mandatory mask wearing (*yes*, *no*), mandatory indoor ventilation (*yes*, *no*), and perception of COVID-19 as a threat (*yes*, *no*). Further, the reliability of information, that they received, pertaining to COVID-19 was measured on a 10-point scale by source (very reliability: 10-point), such as media, government, and family/friends.

### 2.4 Statistical analysis

We aimed to examine COVID-19 phobia among undergraduate and graduate students in Korea, Japan, and China and identify its influencing factors. To this end, we performed the statistical analysis as follows. First, descriptive statistical analysis was performed for each category of COVID-19 phobia in each country. Second, differences in COVID-19 phobia between countries based on independent variables were examined using an ANOVA F-test. Third, we used equation (1) to test the difference of each category of independent variables, by country, with the average value of the COVID-19 phobia scale. We visualized the results of these calculations using GraphPad PRISM 9. Fourth, multiple linear regression was performed, setting the COVID-19 phobia scale as a dependent variable to identify the factors affecting COVID-19 phobia in each country. For each model, the correlation between independent variables was verified using multicollinearity, model fit with an F-test, and explanatory power using the adjusted R^2^.

(1) Gap=(COVID-19Phobiascalebyvariables)−(COVID-19Phobiascaleaverage)

### 2.5 Ethical consideration

All components of this survey were approved by the institutional review board (IRB) of Yonsei University in Korea (IRB document number: 1041849-202204-SB-078-01), Shandong University in China (IRB document number: LL20220425). Written informed consent was obtained from all respondents prior to the data collection. Specifically, we highlighted the respondent's right to refuse the survey request on the first page of the online survey form.

## 3 Results

### 3.1 Respondent characteristics

Table 1 gives the composition and characteristics of the respondents. Participants’ experiences in using self-diagnostic kits were higher in Korea than in Japan or China. In terms of individual preventive behavior, the greatest number of respondents avoided crowded places in China, followed by Korea and Japan. Compliance with mandatory social distancing was observed in the order of Korea, China, and Japan.

**Table 1:** Respondent characteristics by country.

	Korea (n = 460)	Japan (n = 248)	China (n = 788)	t/χ^2^ (P)
Sex				
Male	126 (27.4%)	75 (30.2%)	276 (35.0%)	8.2 (.017)
Female	334 (72.6%)	173 (69.8%)	512 (65.0%)	
Age				
Mean ± SD	22.0 ± 3.5	20.3 ± 0.8	22.9 ± 2.5	84.1 (<.001)
Location of residence				
Urban	390 (84.8%)	175 (70.6%)	730 (92.6%)	80.8 (<.001)
Rural	15.2 (15.2%)	73 (29.4%)	58 (7.4%)	
Number of cohabitants				
Mean ± SD	2.3 ± 1.6	2.4 ± 1.4	3.6 ± 2.3	68.8 (<.001)
Experience with self-administered test kits				
Yes	349 (75.9%)	77 (31.0%)	89 (11.3%)	538.0 (<.001)
No	111 (24.1%)	171 (69.0%)	699 (88.7%)	
Avoiding crowded places				
Yes	210 (45.7%)	86 (34.7%)	592 (75.1%)	179.6 (<.001)
No	250 (54.3%)	162 (65.3%)	196 (24.9%)	
Mandatory mask wearing				
Yes	441 (95.9%)	201 (81.0%)	727 (92.3%)	46.7 (<.001)
No	19 (4.1%)	47 (19.0%)	61 (7.7%)	
Mandatory social distancing				
Yes	405 (88.0%)	117 (47.2%)	644 (81.7%)	170.3 (<.001)
No	55 (12.0%)	131 (52.8%)	144 (18.3%)	
Mandatory indoor ventilation				
Yes	337 (73.3%)	159 (64.1%)	653 (82.9%)	41.9 (<.001)
No	123 (26.7%)	89 (35.9%)	135 (17.1%)	
COVID-19 is not a threat				
Yes	265 (57.6%)	79 (31.9%)	220 (27.9%)	113.3 (<.001)
No	195 (42.4%)	169 (68.1%)	568 (72.1%)	
COVID-19 phobia scale				
Psychological factors	17.2 ± 6.2	17.3 ± 5.5	17.3 ± 6.0	0.1 (.911)
Psychosomatic factors	8.7 ± 4.3	9.2 ± 4.6	8.7 ± 5.1	1.2 (.297)
Economic factors	13.0 ± 4.1	12.9 ± 3.9	10.7 ± 4.1	53.7 (<.001)
Social factors	9.4 ± 4.1	11.1 ± 3.9	13.1 ± 5.1	94.1 (<.001)
Sum	48.1 ± 16.0	50.5 ± 16.1	49.9 ± 17.7	2.1 (.123)
Reliability of information pertaining to COVID-19 (media)				
Mean ± SD	7.1 ± 1.6	6.5 ± 1.8	7.6 ± 1.7	1.3 (.265)
Reliability of information pertaining to COVID-19 (government)				
Mean ± SD	8.0 ± 1.6	7.7 ± 1.9	8.4 ± 1.8	32.1 (<.000)
Reliability of information pertaining to COVID-19 (family/friends)				
Mean ± SD	6.4 ± 1.9	6.1 ± 1.8	7.0 ± 1.9	357.5 (<.000)

**Fig. 1:**
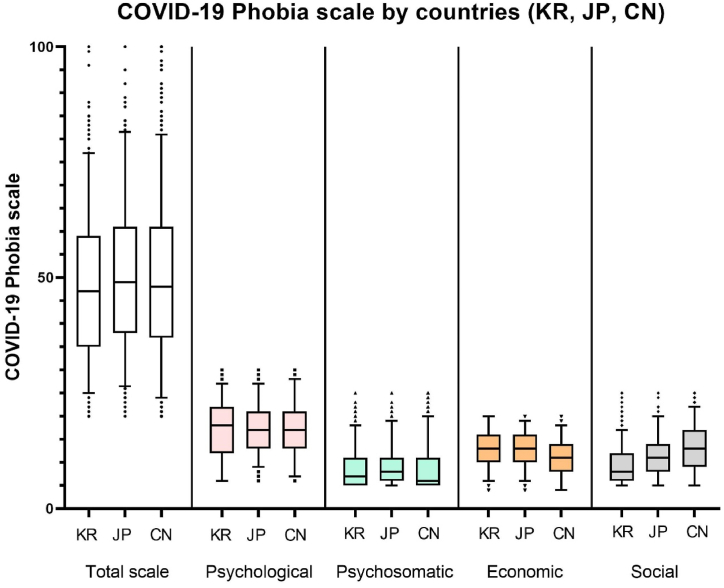
COVID-19 phobia scale by country (KR, JP, CN).

The mean COVID-19 phobia score was highest in Japan at 50.5 points. Psychological fear was identically prevalent in Japan and China, at an average of 17.3 points. Psychosomatic fear was highest in Japan at 9.2 points. Further, economic fear was highest in Korea at 13 points, whereas social fear was highest in China at 13.1 points (Fig. 1).

### 3.2 Analysis of differences in COVID-19 phobia

The presence or absence of characteristic differences in COVID-19 phobia were differentially analyzed by country (Table 2). We observed significant differences in the following categories: men (p = .004), previous experience in using a self-administered test kit (p < .000), not avoiding crowded places (p = .003), not mandating social distancing (p < .000), and perception of COVID-19 as a threat (p = .014). COVID-19 economic and social phobia demonstrated statistically significant differences between countries across all characteristic variables.

**Table 2:** Analysis of differences in COVID-19 phobia according to respondent characteristics.

Variables	COVID-19 Phobia																			
					Psychological				Psychosomatic				Economic				Social			
	KR	JP	CN	P	KR	JP	CN	P	KR	JP	CN	P	KR	JP	CN	P	KR	JP	CN	P
Sex																				
Male	43.8	51.5	49.6	.004	15.4	17.7	16.8	.024	8.1	9.9	9.5	.018	11.8	12.2	10.5	.001	8.5	11.7	12.8	.000
Female	49.7	50.1	50.1	.965	17.9	17.2	17.6	.468	8.9	9.0	8.3	.159	13.4	13.2	10.9	.000	9.7	10.8	13.2	.000
Location of residence																				
Urban	48.2	51.2	49.7	.137	17.2	17.5	17.3	.841	8.7	9.4	8.6	.148	13.0	13.0	10.7	.000	9.4	11.3	13.1	.881
Rural	47.5	48.7	51.5	.348	17.1	16.9	17.4	.881	8.5	8.7	9.9	.156	12.8	12.6	11.0	.021	9.1	10.6	13.2	.000
Experience with self-administered test kits																				
Yes	48.1	50.4	56.3	.000	17.1	17.0	18.9	.045	8.7	9.3	9.9	.071	12.9	12.8	11.9	.126	9.4	11.2	15.6	.000
No	48.2	50.5	49.1	.486	17.3	17.5	17.1	.779	8.5	9.1	8.6	.323	13.1	12.9	10.6	.000	9.3	11.0	12.8	.000
Avoiding crowded places																				
Yes	53.0	58.1	51.2	.230	19.0	19.7	17.8	.749	9.4	11.2	8.9	.671	14.4	14.5	11.1	.000	10.1	12.7	13.4	.000
No	44.1	46.5	46.0	.003	15.6	16.1	15.8	.003	8.0	8.2	8.4	.000	11.7	12.0	9.6	.000	8.8	10.2	12.2	.000
Mandatory mask wearing																				
Yes	48.4	50.9	49.8	.129	17.3	17.6	17.4	.120	8.6	9.1	8.5	.126	13.1	13.1	10.8	.046	9.4	11.0	13.1	.002
No	42.4	48.9	50.8	.185	13.7	16.3	16.3	.895	8.8	9.6	11.0	.300	10.3	11.9	10.2	.000	9.5	11.1	13.3	.000
Mandatory social distancing																				
Yes	48.7	56.3	50.4	.240	17.4	19.2	17.6	.778	8.7	10.8	8.7	.072	13.1	13.8	10.9	.000	9.5	12.4	13.2	.000
No	44.2	45.3	47.7	.000	15.7	15.6	16.1	.011	8.2	7.8	9.0	.000	11.5	12.0	10.1	.000	8.7	9.8	12.5	.000
Mandatory indoor ventilation																				
Yes	49.1	53.1	50.1	.120	17.5	18.2	17.4	.208	8.7	9.7	8.7	.535	13.3	13.3	10.8	.000	9.6	11.8	13.2	.000
No	45.5	45.7	48.9	.054	16.3	15.7	17.1	.272	8.4	8.2	8.8	.049	12.1	12.1	10.3	.000	8.7	9.7	12.8	.000
COVID-19 is a threat																				
Yes	51.7	52.0	50.1	.014	19.0	18.0	17.0	.112	9.2	9.3	9.2	.064	13.6	13.4	10.4	.000	9.9	11.2	13.5	.000
No	45.5	47.3	49.8	.197	15.9	15.8	17.5	.008	8.2	9.0	8.5	.075	12.5	11.7	10.5	.000	9.0	10.8	10.9	.000

**Fig. 2:**
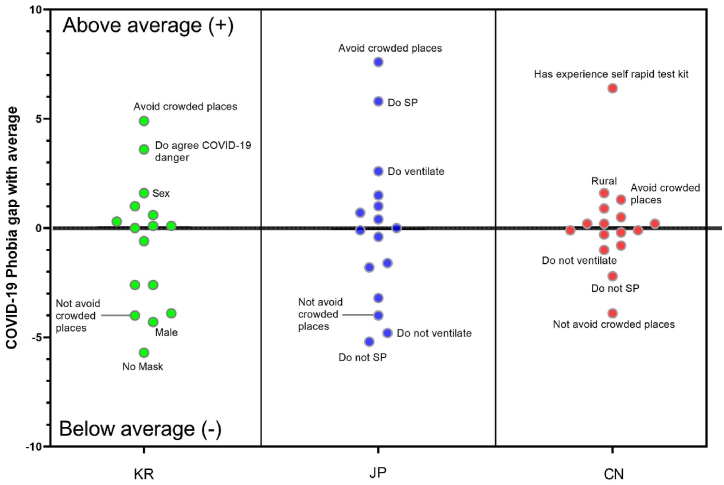
COVID-19 phobia gap with average by each country.

Table 3 and Fig. 2 shows the mean standard values for the differences in characteristic COVID-19 phobia in Korea, Japan, and China. The top three characteristics that demonstrated the highest level of COVID-19 phobia in Korea were *avoiding crowded places* (4.87), *perceiving COVID-19 as a threat* (3.57), and *female* (1.57). Meanwhile, the three characteristics that demonstrated the lowest level were *non-compliance with mask wearing* (−5.73), *male* (−4.33), and *not avoiding crowded places* (−4.03).

**Table 3:** Top 5 of Differences in mean COVID-19 phobia scores by characteristic group.

	Rank	Korea		Japan		China	
		Group	Gap^a^	Group	Gap^a^	Group	Gap^a^
Above average (+)	1	Avoiding crowded places	4.87	Avoiding crowded places	7.61	Has experience self-rapid test kit	6.41
	2	COVID-19 is a threat	3.57	Compliance with social distancing	5.81	Rural	1.61
	3	Female	1.57	Enforcing indoor ventilation	2.61	Avoiding crowded places	1.31
	4	Enforcing indoor ventilation	0.97	COVID-19 is a threat	1.51	Compliance with mask wearing	0.91
	5	Compliance with social distancing	0.57	Male	1.01	Compliance with social distancing	0.51
Below average (−)	1	Non-compliance with mask wearing	−5.73	Non-compliance with social distancing	−5.18	Not avoiding crowded places	−3.88
	2	Male	−4.33	Not enforcing indoor ventilation	−4.78	Non-compliance with social distancing	−2.18
	3	Not avoiding crowded places	−4.03	Not avoiding crowded places	−3.98	Not enforcing indoor ventilation	−0.98
	4	Non-compliance with social distancing	−3.93	COVID-19 is not a threat	−3.18	No previous experience with self-administered test kits	−0.78
	5	COVID-19 is not a threat	−2.63	Rural	−1.78	Male	−0.28

a Gap= (COVID-19 Phobia scale by variables) − (COVID-19 Phobia scale average).

In Japan, the top three characteristics demonstrating COVID-19 phobia were *avoiding crowded places* (7.61), *complying with social distancing* (5.81), and *enforcing indoor ventilation* (2.61). The three characteristics demonstrating the lowest level were *non-compliance with social distancing* (−5.18), *not enforcing indoor ventilation* (−4.78), and *not avoiding crowded places* (−3.98).

Finally, in China, the top three characteristics demonstrating COVID-19 phobia were *previous experience with self-administered test kits* (6.41), *rural* (1.61), and *avoiding crowded places* (1.31). The three characteristics demonstrating the lowest level were not *avoiding crowded places* (−3.96), *non-compliance with social distancing* (−2.18), and *not enforcing indoor ventilation* (−0.98).

### 3.3 COVID-19 phobia linear regression analysis

We performed a linear regression analysis to examine the factors affecting COVID-19 phobia in each country (Table 4). In Korea, COVID-19 phobia scores were significantly higher among women than in men (β = 5.135, p = .002), whereas scores were significantly lower when there was no previous experience with self-administered testing kits (β = −1.748, p < .000). University students who were avoiding crowded places had significantly higher scores (β = 7.481, p < .000), as did those who perceived COVID-19 as a threat (β = 4.928, p = .001). In Japan, COVID-19 phobia scores were significantly higher in individuals who were avoiding crowded places (β = 8.407, p < .000) and those who complied with social distancing mandates (β = 8.476, p < .000). In China, a lack of previous experience with self-administered testing kits was associated with significantly lower phobia scores (β = −7.164, p < .000). Individuals who were avoiding crowded places had significantly higher scores (β = 5.036, p = .002). Indeed, COVID-19 phobia scores rose significantly, by 1.864 points, for each point added to the reliability of information pertaining to COVID-19 (family/friends). We tested the model fit of the linear regression analysis using the F-test. The fit was found to be sufficient for all countries at a p-value of less than 0.05.

**Table 4:** Results of linear regression analysis for factors influencing COVID-19 phobia by country.

Variables	Korea			Japan			China		
	β	t	P	β	t	P	β	t	P
Sex									
Male	1			1			1		
Female	5.135	3.164	.002**	−2.019	−.960	.338	.719	.545	.586
Location of residence									
Urban	1			1			1		
Rural	−1.318	−.659	.510	−2.316	−1.085	.279	1.824	.771	.441
Experience with self-administered test kits									
Yes	1			1			1		
No	−1.748	−1.031	.000***	.042	.020	.984	−7.164	−3.655	.000***
Avoiding crowded places									
No	1			1			1		
Yes	7.481	4.935	.000***	8.407	3.981	.000***	5.036	3.121	.002**
Mandatory mask wearing									
No	1			1			1		
Yes	.466	.120	.905	−1.524	−.605	.546	−4.123	−1.450	.147
Mandatory social distancing									
No	1			1			1		
Yes	1.908	.814	.416	8.476	3.882	.000***	.512	.260	.795
Mandatory indoor ventilation									
No	1			1			1		
Yes	1.831	1.092	.275	.541	.241	.810	−1.016	−.543	.588
COVID-19 is a threat									
No	1			1			1		
Yes	4.928	−.080	.001**	2.957	1.416	.159	−.853	−.596	.551
Reliability of information pertaining to COVID-19 (media)	−.047	−1.044	.936	.994	1.247	.214	.216	.463	.644
Reliability of information pertaining to COVID-19 (government)	−.607	.376	.297	−.360	−.529	.598	−.592	−1.377	.169
Reliability of information pertaining to COVID-19 (family/friends)	.153	3.336	.707	.608	1.002	.318	1.864	4.938	.000***
Age	4.928	1.367	.172	−1.838	−1.611	.109	.162	.643	.520
Constant	27.268			75.810		.003	.162		
F	5.529***			5.521***			5.241***		
R^2^	.129			.220			.075		

## 4 Discussion

This study aimed to examine and identify the factors influencing characteristic differences in COVID-19 phobia among undergraduate and graduate students in Korea, Japan, and China. Scores from the COVID-19 phobia scale were similar across the three countries (Japan, 50.5 points; China, 49.9 points; Korea, 48.1 points).

Among the four types of fear listed on the COVID-19 phobia scale, scores for economic fear were found to be higher in Korea (13.0 points) and Japan (12.9 points) by approximately 2 points compared with China (10.7 points). Economic fear increases when income is reduced owing to restrictions on economic activities from changes in the external environment [15]. As business hours and incomes of individual business owners decreased following the spread of COVID-19, so did the rate of part-time employment [16]. The part-time employment rates of undergraduate and graduate students were revealed to be higher in Korea (70%) compared with Japan (60.7%) and China (43%) [17,18]. Thus, more Korean undergraduate students held part-time jobs to support their living expenses, tuition, and ancillary fees compared with the other two countries. Nonetheless, with the spread of COVID-19, the Korean government enforced measures that restricted the activities of individual business owners, such as social distancing and reduced business hours, which are presumed to have led to financial challenges for students seeking part-time employment [[19], [20], [21]]; Economou et al., 2021). Conversely, a Chinese study reported that Chinese university students felt financial strain owing to tuition because of the COVID-19 pandemic [12,22]. As for Chinese students, little of them had part-time job, so the fear should be lower, but they may worry about their parents’ job during the pandemic.

Social fear, rated out of 25 points, was reported to be higher in China (13.1 points) than in Japan (11.1 points) and Korea (9.4 points). Social fear stems from the societal perception and restrictions that follow COVID-19 infection. The Chinese government enforced stricter control over the prevention of COVID-19 and the management of confirmed cases compared with neighboring countries [23]. Such strict control can have a positive effect of enabling the thorough management and control of confirmed cases. Indeed, the number of confirmed cases in China is lower than in Korea or Japan [[24], [25], [26]]. According to a study that analyzed the effect of governmental control on university students across nine countries [27], more restrictive governmental control could worsen students’ mental health; related studies have provided evidence for stress and phobia [28,29]. Although strict government controls possible to raise fear, they are effective for the control of COVID-19, highlighting the need for comprehensive evidence on the appropriate level of control. Japan is a country that has not experienced a SARS epidemic unlike Korea and China. There was a lack of preparation to deal with COVID-19, and even citizens are less accustomed to the spread of the epidemic than in Korea and China. Therefore, it is thought that fear has arisen due to the social spread of COVID-19 [30].

We compared the characteristics of the Korean, Japanese, and Chinese survey respondents based on a calculation of differences to the average scores of COVID-19 phobias, by category. Data from all three countries demonstrated higher than average levels of phobia among respondents who complied with COVID-19 prevention measures regarding crowded places, indoor ventilation, social distancing, and mask wearing. This was consistent with previous findings that suggested a correlation between COVID-19 phobia and preventive behavior [31]; and Griffiths, 2020; [32,33]. University students in Japan demonstrated particularly higher than average levels of phobia among those who complied with COVID-19 prevention measures. This may be associated with the Japanese tendency of policy credence and compliance in emergency situations. According to a previous study, Japanese people demonstrate a tendency to trust and comply with the government's guidelines as a priority during crisis situations, such as a pandemic [34]. Our findings may reflect such active compliance with government recommendations for COVID-19 prevention following a heightened sense of fear.

According to the results of the regression analysis, which analyzed the factors influencing COVID-19 phobia, findings regarding COVID-19 phobia can be summarized as follows. First, we found an association between the tendency to avoid crowded places and COVID-19 phobia. All three governments—Korea, Japan, and China—enforced policies that emphasized social distancing against the droplet transmission of COVID-19. Thus, the policies enforced in the three countries clearly established, among their citizens, that contact with others must be restricted to prevent the spread of COVID-19. Individuals with high fear were those who avoided crowded places to minimize close contact.

Second, mask wearing and indoor ventilation mandates were not found to be significantly correlated with COVID-19 phobia in all three countries. Mask wearing was mandated in all countries, and the respondents indicated a compliance rate of over 90%, which can be interpreted as independent of COVID-19 phobia. Nevertheless, despite the regular emphasis and promotion of the need for and importance of indoor ventilation at the government level, many students were found not to be observing proper indoor ventilation in their respective settings. The associations that were not significant with COVID-19 phobia suggest the need for more active promotion of the need for and importance of indoor ventilation as a means to prevent COVID-19.

Third, the presentation of COVID-19 information by media and governmental institutions did not have a significant effect on reducing COVID-19 phobia in Korea, Japan, or China. All three countries had a 90% rate (high) of smartphone use, alongside well-developed nationwide smartphone internet infrastructure [35]. Indeed, citizens of developed countries independently acquire COVID-19 information through the internet, YouTube, Twitter, and other social media platforms. Nevertheless, our findings revealed that contrary to Korean or Japanese students, Chinese students demonstrated a significantly higher level of COVID-19 phobia when they acquired information related to COVID-19 from their family or friends. A greater proportion of Chinese students lived with their families or friends compared with Korean and Japanese students, which may imply more conversations surrounding COVID-19 at home. In such instances, individuals may share small and provocative pieces of information out of concern for COVID-19, which may include false information [36]. An explanation is that individuals tend to accept and trust COVID-19 information acquired from family, friends, or acquaintances more actively compared with information from the internet or social media [37]. This, in turn, holds high potential to create a sense of fear.

Our study has a few limitations. First, the survey periods in the three countries were different. This difference could have affected the degree to which the results reflected the pattern of the spread and global trend of COVID-19. Second, a non-sampling error may have occurred owing to the use of online surveys. We conducted online surveys owing to the rapid spread of COVID-19, in addition to geographical reasons. Online surveys are known to generate more non-sampling errors than face-to-face surveys [38]. Third, the possibility that the levels of COVID-19 phobia were measured as higher than usual must be considered, as the spread of COVID-19 had rapidly increased during the survey periods.

## 5 Conclusion

We examined the characteristic differences and influencing factors of COVID-19 phobia among Korean, Japanese, and Chinese undergraduate and graduate students. The levels of COVID-19 phobia were similar across Korea, Japan, and China. However, economic fear was the most common among Korean and Japanese university students, whereas social fear was most common among Chinese university students. Furthermore, university students in all three countries demonstrated greater COVID-19 phobia if they were complying with COVID-19 prevention measures. His implies that students in the three countries knew that it was necessary to comply with COVID-19 preventive behaviors to prevent infections.

Mental health issues caused by COVID-19 must be reflected in the policies enforced by governments in the post-COVID era. Thus, our findings may be referenced when establishing an approach strategy to reduce COVID-19 phobia among Chinese, Japanese, and Korean students.

## Author contribution statement

Hocheol Lee; Fanlei Kong; Myo Nyein Aung; Yoshihisa Shirayama; Eun Woo Nam: Conceived and designed the study; Performed the experiments; Analyzed and interpreted the data; Contributed reagents, materials, analysis tools or data; Wrote the paper.

Moto Yuasa; Bo Zhao; Mahyeon Kim: Conceived and designed the study.

## Funding statement

This work was supported by the National Research Foundation of Korea grant funded by the Korea government (MSIT) [NRF-2021R1C1C2005464].

## Data availability statement

Data will be made available on request.

## Declaration of interest’s statement

The authors declare no competing interests.
