# Supplementary material for: COVID-19 Phobia among Korean, Chinese, and Japanese students: An international comparative study
Source: Heliyon. 2023 Apr 7;9(4):e15275. doi: 10.1016/j.heliyon.2023.e15275 (PMC10081880; doi:10.1016/j.heliyon.2023.e15275)
Supplement: Multimedia component 1 [file mmc1.pdf]

Survey on COVID-19 (2022)

[PART 1] Questions about COVID-19 infection

N	Questions	Answers	Remarks
A1	Have you ever been infected with COVID-19?	1. Yes (Go to next question)<br>2. No (→ PART 2)	
A2	('Yes' to question 1) When did you get infected with COVID-19?	Year ______<br>Month ___ Day ____	
A3	Where do you think you got infected with COVID-19?	1. School<br>2. Academy<br>3. Home<br>4. Public places<br>5. Restaurant<br>6. Other (______________)<br>7. I don't know.	
A4	Who do you think you got COVID-19 from?	1. Family<br>2. Friends<br>3. Cohabitant (person who you are living with)<br>4. Coworkers<br>5. 3rd person that I don't know<br>6. Other (__________)	
A5	Were you wearing a mask at the time of infection?	1. Yes<br>2. No<br>9. I don't know.	

A6	Please select all the main symptoms you experienced when you were infected with COVID-19 (You can choose multiple responses).	1. Cough<br>2. Sore throat<br>3. Headache<br>4. Nausea<br>5. Diarrhea<br>6. Abdominal pain<br>7. Chest pain<br>8. runny nose<br>9. phlegm<br>10. Loss of taste<br>11. Loss of Smell<br>12. Lethargy<br>13. Memory Loss<br>14. Other (__________)	
A7	Do you think you have after-effect after getting infected with COVID-19?	1. Yes (→ Question A7)<br>2. No (→ Question A8)	
A8	If you have after-effect, please select all symptoms. (multiple responses)	1. Runny nose<br>2. Memory loss<br>3. Lethargy<br>4. Loss of taste<br>5. Loss of Smell<br>6. Myalgia (muscle pain)<br>7. Mental trauma<br>8. Other (___________)	
A9	Please select where you received your COVID-19 test. (multiple responses)	1. Primary/Secondary Hospital<br>2. Public health center<br>3. University Hospital<br>4. Self-Kit<br>5. Other (______)	
A10	What body part did you test through when testing for COVID-19?	1. Both mouth and nose<br>2. Mouth<br>3. Nose	
A11	In which place you got confirmed as COVID-19 positive?	____ city (county)<br>____ gu (eup)<br>____ dong (myeon)	
A12	What type of house do you currently live in?	1. Apartment<br>2. Multi-family house/Villa	

		3. One-room<br>4. Detached house<br>5. Dormitory	
A13	Where did you quarantine after being confirmed as COVID-19 positive?	1. Current Residence<br>2. Quarantine facility<br>3. Another place<br>(________)	
A14	Did you have anyone stayed with you during self-quarantine? (family, acquaintances, friends, etc.)	1. Yes<br>2. No	
A15	What is the total number of days of your self-quarantine?	Total ________ days	
A16	Have you been prescribed any medications since you were diagnosed as COVID-19 infected?	1. Yes<br>2. No	
A17	(If you were prescribed medication) How did you get your medication?	1. Picked up directly from the pharmacy<br>2. Via delivery<br>3. Received through other person (family, friend, etc.)<br>4. Other (__________)	

[PART 2] COVID-19 Prevention / Information

N	Items	Answers
B1	Have you ever had a COVID-19 PCR test?	1. Yes<br>2. No
B2	What is your vaccination status?	1. Vaccinated (up to 1st dose)<br>2. Vaccinated (up to 2nd dose)<br>3. Vaccinated (up to 3rd dose)<br>4. Vaccinated (up to 4th dose)<br>5. Not vaccinated
B3	Have you ever seen or heard of anyone who got confirmed as COVID-19 positive, but do outside activities without self-quarantine?	1. Yes<br>2. No
B4	Do you think there are people confirmed as COVID-19 positive around you without self-quarantine?	1. Yes<br>2. No
B5	Do you think the government's social distancing policy is effective?	1. I don't think it is effective.<br>2. I think it is little effective.<br>3. I think it is very effective.
B6	Do you agree with the government's COVID-19 mitigation policy?	1. I don't agree<br>2. I agree.
B7	Have you ever used a self-test kit?	1. Yes<br>2. No
B8	How often did you use the self-test kit?	______ times per week

B9	To what extent do you think wearing a mask is effective in preventing COVID-19? (out of 10)	______ points / 10 points<br><br>*0 point: Not effective at all<br>10 points: Strongly effective					
B10	How often do you wear a mask in public places? (out of 10)	______ points / 10 points<br><br>*0 point: not wear at all<br>10 points: always wear					
B11	Do you wear a mask when you are with your family at home? (out of 10)	______ points / 10 points<br><br>*0 point: not wear at all<br>10 points: always wear					
B12	How often do you use the sources below to get information about COVID-19?		Very rarely	Some times	Usually	Often	Very often
	B15-1. Internet news		1	2	3	4	5
	B15-2. Conversation with family and friends		1	2	3	4	5
	B15-3. From medical personnel in hospitals and public health centers		1	2	3	4	5
	B15-4. Social media (Facebook, YouTube, KakaoTalk, Instagram, etc.)		1	2	3	4	5
	B15-5. Information sent from public institutions		1	2	3	4	5
	B15-6. TV		1	2	3	4	5
	B15-7. Cellphone		1	2	3	4	5
	B15-8. Radio		1	2	3	4	5
B13	How much do you trust the COVID-19 information provided by the media (Internet/TV)?<br>(out of 10)	______ points / 10 points<br><br>*0 point: do not trust at all<br>10 points: Trust unconditionally					
B14	How much do you trust	______ points / 10 points					

	the COVID-19 information provided by public health centers/public institutions? (out of 10)	*0 point: do not trust at all<br>10 points: Trust unconditionally
B15	How much do you trust the COVID-19 information provided by your family/friends? (out of 10)	______ points / 10 points<br><br>*0 point: do not trust at all<br>10 points: Trust unconditionally
B16	Do you think Omicron COVID-19 is similar to the flu and is not life-threatening?	1. Yes<br>2. No

[PART 3] COVID-19 KAP

	Item	Yes	No	Don't know
Knowledge				
K1	The main clinical symptoms of COVID-19 are fever, fatigue, dry cough, and myalgia.	1	2	9
K2	There currently is no effective cure for COVID-2019, but early symptomatic and supportive treatment can help most patients recover from infection.	1	2	9
K3	Not all persons with COVID-2019 will develop severe cases. Only those who are elderly have chronic illnesses are more likely to be in severe cases.	1	2	9
K4	Eating or contacting wild animals would result in infection by the COVID-19 virus.	1	2	9
K5	The COVID-19 virus spreads via respiratory droplets of infected individuals.	1	2	9
K6	Ordinary residents can wear general medical masks to prevent infection by the COVID-19 virus.	1	2	9

Attitude						
		**Very low**		**Neither low nor high**		**Very high**
A1	What do you think is the possibility of your COVID-19 infection?	1	2	3	4	5
A2	What do you think will be the severity if COVID-19 infects you?	1	2	3	4	5
A3	What do you think will be the severity if COVID-19 infects your family member, acquaintances or friends?	1	2	3	4	5
Practice						
		Never		Usually		Always
P1	Do you avoid visiting crowded places?					
P2	Do you wear facial masks?					
P3	Do you practice social distancing?					

P4	Do you regularly ventilate the indoor place?					

[PART 4] COVID-19 Phobia scale

	item	Strongly disagree	Disagree	Agree	Generally agree	Strongly agree
PH1	The fear of coming down with coronavirus makes me very anxious.	1	2	3	4	5
PH2	I experience stomach-aches out of the fear of coronavirus.	1	2	3	4	5
PH3	After the coronavirus pandemic, I feel extremely anxious when I see people coughing.	1	2	3	4	5
PH4	The possibility of food supply shortage due to the coronavirus pandemic causes me anxiety.	1	2	3	4	5
PH5	I am extremely afraid that someone in my family might become infected by the coronavirus.	1	2	3	4	5
PH6	I experience chest pain out of the fear of coronavirus.	1	2	3	4	5
PH7	After the coronavirus pandemic, I actively avoid people I see sneezing.	1	2	3	4	5

PH8	The possibility of shortages in cleaning supplies due to the coronavirus pandemic causes me anxiety.	1	2	3	4	5
PH9	News about coronavirus-related deaths causes me great anxiety.	1	2	3	4	5
PH10	I experience tremors due to the fear of coronavirus.	1	2	3	4	5
PH11	Following the coronavirus pandemic, I have noticed that I spend extensive periods of time washing my hands.	1	2	3	4	5
PH12	I stock food with the fear of coronavirus.	1	2	3	4	5
PH13	Uncertainties surrounding coronavirus cause me enormous anxiety.	1	2	3	4	5
PH14	I experience sleep problems out of the fear of coronavirus.	1	2	3	4	5
PH15	The fear of coming down with coronavirus seriously impedes my social relationships.	1	2	3	4	5
PH16	After the coronavirus pandemic, I do not feel relaxed unless I constantly	1	2	3	4	5

	check on my supplies at home.					
PH17	The pace that coronavirus has spread causes me great panic.	1	2	3	4	5
PH18	Coronavirus makes me so tense that I find myself unable to do the thing I previously had no problem doing.	1	2	3	4	5
PH19	I am unable to curb my anxiety of catching coronavirus from others.	1	2	3	4	5
PH20	I argue passionately (or want to argue) with people I consider to be behaving irresponsibly in the face of coronavirus.	1	2	3	4	5

Source : Arpaci, Ibrahim, et al. "COVID-19 phobia in the United States: Validation of the COVID-19 Phobia Scale (C19P-SE)." *Death studies* (2020): 1-7.

[PART 5] Respondent Information

N	Item	Answer	Remark
E1	What year were you born?	Year ______	
E2	What is your sex?	1. Male<br>2. Female	
E3	What is your job?	1. University student (1st grade)<br>2. University student (2nd grade)<br>3. University student (3rd grade)<br>4. University student (4th grade and above)<br>5. Graduate student (master's course)<br>6. Ph.D. candidate and above<br>7. Other (__________)	
E4	Where do you currently live in?		
E5	What type of area do you currently live in?	1. Urban<br>2. Rural	
E6	Who do you live with?	1. Family<br>2. Friend<br>3. Other (_________)	
E7	How many people do you live with?	______ people	
E8	What is your nationality?		
E9	What is your major?	____________	
E10	What is your religion?	1. Christian (Protestantism)<br>2. Buddhism<br>3. Catholic<br>4. Muslim<br>5. Other (__________)	
